# ASO Author Reflections: What is the Quality of Reporting Patient-Reported Outcome Measures in Locally Recurrent Rectal Cancer?

**DOI:** 10.1245/s10434-023-13503-6

**Published:** 2023-04-21

**Authors:** Niamh Aine McKigney, Fergus Houston, Ellen Ross, Galina Velikova, Julia M. Brown, Deena P. Harji

**Affiliations:** 1 Clinical Trials Research Unit, Leeds Institute of Clinical Trials Research, University of Leeds, Leeds, UK; 2 Princess Alexandra Hospital, Brisbane, Australia; 3 Inverclyde Royal Hospital, Greenock, Scotland UK; 4 Leeds Institute of Medical Research at St James’s, University of Leeds, Leeds, UK; 5 St. James’s Institute of Oncology, St. James’s University Hospital, Leeds, UK; 6 Department of Colorectal Surgery, Manchester University NHS Foundation Trust, Manchester, UK

## Past

There is increasing interest in assessing patient-reported outcomes, such as health-related quality of life, in locally recurrent rectal cancer (LRRC). Emphasising the importance of patient-centred outcome reporting to guide decision-making in this cohort of patients. This can only be achieved with the availability of high-quality patient-reported outcome data. Previous reviews have highlighted several limitations regarding studies reporting patient-reported outcomes in LRRC, including variability in the patient-reported outcome measures (PROMs) being used, and in the timing of PROM assessments, with the evidence being low in quality overall.^1–4^ PROMs reported in LRRC have not previously been assessed against the COnsensus-based Standards for the selection of health Measurement INstruments (COSMIN) Risk of Bias checklist.^5^

## Present

This systematic review focused on assessing the methodological quality of studies reporting PROMs in LRRC to identify the PROMs being used and to assess their psychometric properties using the COSMIN Risk of Bias checklist.^6^ The results identified several issues related to the quality of reporting of PROMs in LRRC, most importantly that none of the PROMs currently being used to report outcomes in LRRC have been validated for use in this group of patients. Methods for handling missing PROM data and defining the patient-reported outcome of interest are also poorly reported. However, the person completing the PROM and method of data collection, and conclusions and discussion of the clinical relevance of the patient-reported outcome data, were well reported.

## Future

The lack of disease-specific PROMs which have been validated for use in LRRC, is the most important issue to address to improve the quality of reporting PROMs in LRRC. Several approaches could be employed, including developing new disease-specific PROMs for patients with LRRC or undertaking content validity studies of the PROMs which are currently being used in LRRC.
